# Optimization and quality evaluation of infrared‐dried kiwifruit slices

**DOI:** 10.1002/fsn3.1253

**Published:** 2020-01-01

**Authors:** Ebrahim Sadeghi, Ali Haghighi Asl, Kamyar Movagharnejad

**Affiliations:** ^1^ Faculty of Chemical, Petroleum and Gas Engineering Semnan University Semnan Iran; ^2^ Faculty of Chemical Engineering Babol Noshirvani University of Technology Babol Iran

**Keywords:** infrared drying, kiwifruit, rehydration, response surface methodology, shrinkage

## Abstract

Infrared drying characteristics of kiwifruits under natural and forced drying air convection with different conditions were investigated. An experimental study along with statistical analysis aimed to evaluate quality characteristics of infrared‐dried kiwifruit slices, in terms of drying time, rehydration ratio and shrinkage as a function of infrared power levels, slice thicknesses, slice distance from the infrared lamps, and air velocity. Response surface methodology was used for optimization of drying parameters with employing desirability function. Minimum drying time, shrinkage, and maximum rehydration ratio assumed as criteria for optimizing drying conditions of kiwifruit slices were strongly dependent on the drying conditions. All operating variables had a significant effect on total responses, but slice thickness almost was the most prominent factor. The slices dried at the highest power level, the lowest distance from the Infrared lamp, the least thickness, and air velocity showed a higher rehydration capacity than slices dried at the other conditions.

## Graphical abstract

The results of this research can provide a theoretical and practical basis for further design, optimization, and scale‐up of such processes and also the development of models that predict the behavior of infrared food drying.

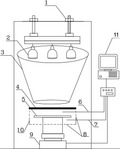

## 1 INTRODUCTION

Today, the growing reduction of the use of chemical for food preservation and high rate of the popularity of high‐quality fast‐dried products with good rehydration attributes have led to renewed interest in drying (Maskan, 2001).

Owing to the low thermal conductivity of the high sugar‐containing food such as kiwifruit, heat transfer inside the inner sections of it in the falling rate period is restricted during conventional heating, which causes prolonged drying time, reduction in its physicochemical properties, and low energy efficiency (Kocabiyik, Yilmaz, Tuncel, Sumer, & Buyukcan, 2015).

During drying, a pressure unbalance produced between the inner of the material and the external pressure by eliminating moisture from the material generates contracting stresses that cause to material shrinkage (Mayor & Sereno, 2004). Rehydration, as total or partial reconstitution of water, is a criterion of the injuries to the material caused by drying (Lewicki, 1998) and to a large extent determining the final quality of the product (Mohammadi, Rafiee, Keyhani, & Emam‐Djomeh, 2008).

The processing conditions are one of the factors affecting the magnitude of shrinkage. Khraisheh, Cooper, and Magee (1997) have reported that the increase in air velocity produces less shrinkage. Prolonged exposure to high drying temperature may lead to considerable degradation of quality characteristics (Zhang, Tang, Mujumdar, & Wang, 2006).

Infrared is a suitable heating method for the production of high‐quality‐dried foods at low cost to decrease the drying time and as well as appropriate for thin layers drying of samples (Doymaz, 2014).

Some authors have illustrated that the drying time reduced significantly with increase in IR power. Some other authors found that infrared drying system is able to give better qualities of a product such as color and shrinkage (Ponkham, Meeso, Soponronnarit, & Siriamornpun, 2012).

By maximizing rehydration and minimizing operational parameters, drying time and specific energy have found the optimum drying conditions (Kocabiyik & Tezer, 2009).

Response surface methodology (RSM) employs to forecast multivariate statistical model equations for concurrent multiple optimization studies and allows an experimenter to make efficient detection of a process or system (Aghilinategh, Rafiee, Hosseinpour, Omid, & Mohtasebi, 2015).

Despite several experimental studies conducted with different drying system including investigation of quality characteristics (Maskan, 2001), simulation of process (Chen, Pirini, & Ozilgen, 2001), and modeling of the drying kinetics of kiwifruits (Simal, Femenia, Garau, & Rosselló, 2005), little data currently exist on the processing of fresh kiwifruit with IR drying system. Concerning the appropriate combinations of IR power level (*IP*), slice thickness (*λ*), slice distance from the IR lamps (Δ), and drying air velocity (V), for optimum responses in IR dryer, almost studies have not been performed up to now. Therefore, the aim of our study was to investigate the IR drying of kiwifruit slices under natural and forced drying air convection modes with respect to drying kinetics, shrinkage, and rehydration and to relate operating variables in a mathematical equation and to optimize the drying conditions of kiwifruit slices regarding to the quality parameters to determine acceptable product quality in IR drier.

## 2 MATERIALS AND METHODS

### 2.1 Materials

Kiwifruits were prepared from a local market in Amol, Iran. In order to decelerate the respiration, physiological, and chemical changes (Mohammadi, Rafiee, Keyhani, & Emam‐Djomeh, 2009), all samples were stored in a refrigerator at 4 ± 0.5°C for at least 48 hr. Prior to drying process, samples were placed outside of the refrigerator for about an hour to reach room temperature and then peeled and sliced into 2, 4, and 6 mm thick and about 40 mm diameter. The moisture content of slices before drying was measured by a moisture analyzer (A&D Company). The initial moisture content found to be around 4.7 g water/g dry solid (d.b.). The drying tests were carried out down to a final moisture content of about 0.20 d.b. similar to that reported for kiwifruit (Diamante, Durand, Savage, & Vanhanen, 2010; Doymaz, 2009).

### 2.2 The experimental equipment and procedures

Thin‐layer drying of kiwifruit slices was done in a laboratory scale single‐tray IR dryer at different conditions that was designed and made at the Babol Noshirvani University of Technology, Iran.

**Figure 1:**
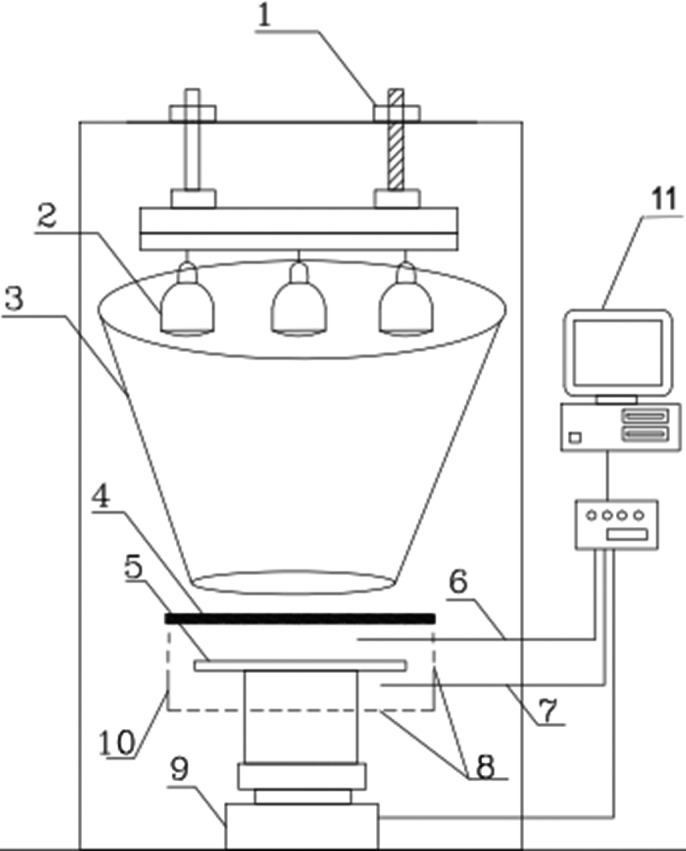
Laboratory IR dryer. 1—distance adjustment screws, 2—IR lamps, 3—cone cover, 4— quartz glass, 5—aluminum tray, 6—psychrometer, 7—anemometer, 8—holes, 9—balance, 10—drying chamber, and 11—computer

The drying chamber included an aluminum tray and an entrance door for loading and unloading the tray. Airflow can easily enter into drying chamber through holes at the bottom of the dryer and leave as natural convection through some holes provided on the two opposite walls of it. To leave airflow as forced convection, the drying chamber is replaced with a similar one equipped with a fan located on one of the walls. It is powered by a variable speed controller. Air velocity was measured by an anemometer (Airflow Development., LTD, UK) with an accuracy of ±0.1 m/s. IR lamps (4–8 × 250 W) were completely surrounded with an external cover (Radiation loss can be negligible) whose inner surfaces were covered by thick aluminum foil to reflect radiation toward slices on the tray and to provide thermal energy (Figure 1).

The slices were uniformly distributed on the tray and were heated to high temperature inside the chamber and then air along with moisture vapors escape through the side ventilation holes or a fan depending on the type of experiments.

To obtain the drying curves, moisture loss was continuously recorded by using a digital electronic balance of ±0.1 g accuracy (A&D Company, Japan). Prior to the drying experiments to ensure steady‐state in tests, the IR dryer was running without any slice for about 80–100 min.

### 2.3 Mathematical modeling of drying

Drying curves provide very important information with respect to the mechanisms of moisture movement (Seremet, Botez, Nistor, Andronoiu, & Mocanu, 2016). Equation (1) shows the moisture content of slices at any time of drying (M_t_, g water/g dry solid) (da Silva, Precker, & Lima, 2009):

(1) $$M_{t}=\frac{W_{t}-W_{\mathrm{dm}}}{W_{\mathrm{dm}}}$$

where, W_t_, and W_dm_ are the weights of the kiwifruit slices at any time of drying and the dry solid of them (g), respectively.

Moisture ratio (MR) represents the existing moisture content at any time in the kiwifruit slices to the amount of initial moisture and was calculated using Equation (2):

(2) $$MR=\frac{M_{t}-M_{e}}{M_{0}-M_{e}}$$

Due to the relatively small equilibrium moisture content (M_e_) compared to initial moisture content (M_0_) at IR drying process (M_e_), is considered zero, so MR can be rewritten as follows (Özdemir, Aktaş, Şevik, & Khanlari, 2017):

(3) $$MR=\frac{M_{t}}{M_{0}}$$

The drying rate (DR) of slices was calculated according to Equation (4) (Doymaz, 2014):

(4) $$DR=\frac{M_{t}-M_{t+\Delta t}}{\Delta t}$$

where M*_t_*_+Δ_ *_t_* is moisture content at *t*+Δ*t*, and Δ*t* is the time interval (min).

### 2.4 Quality evaluation of kiwifruit slices

#### 2.4.1 Rehydration ratio

Generally, it found that the greater the drying, the slower and less complete is the degree of rehydration (Mujumdar, 2014). At the end of each run, rehydration test was performed by immersing a certain weight of slice inside a 100‐ml beaker containing distilled water at 60°C using a water bath for 30 min (Chakraborty, Mukhopadhyay, Bera, & Suman, 2011). After completing the rehydration period, the slice was then taken out, drained on a metal sieve, blotted with soft tissue paper to remove excess water on the slice surface, and finally weighed using an electronic digital balance with accuracy of 0.001 g (GF1000, A&D company).

With regard to recorded weights of the slice before and after the rehydration, the rehydration ratio (RR) was calculated as follows (Maskan, 2001):

(5) $$RR=\frac{W_{r}}{W_{d}}$$

where W_r_ and W_d_ are the weights of IR‐dried kiwifruit slices after and before rehydration (g), respectively.

#### 2.4.2 Shrinkage

The shrinkage (Sh) percentage was calculated by Equation (6) (Ciurzyńska, Lenart, & Kawka, 2013):

(6) $$\%Sh=\left(1-\frac{V_{d}}{V_{0}}\right)\times 100$$

where *V*_d_ and *V*_0_ are the superficial volume of a kiwifruit slice after and before drying (ml), respectively.

Solvent displacement is one of the usual methods for measurement of the sample volume (Ciurzyńska et al., 2013). The used solvent has to be organic and does not interact with the components of the sample. In order to the complete immersion, the sample has to have a higher density than the solvent (Ruhanian & Movagharnejad, 2016). Therefore, solvents such as toluene or n‐heptane are appropriate for the measurement of the sample volume. The slice volume was calculated as following (McMinn & Magee, 1997):

(7) $$V=V_{c}-\frac{M_{t}-M_{C}-M}{\rho_{s}}$$

where *V*_c_, *M_t_*, *M_c_*, *M* and *ρ_s_* are the glass graduated cylinder volume, total mass of the cylinder, slice, and toluene, mass of the cylinder, the mass of the slice, and the toluene density, respectively.

### 2.5 Experimental design and optimization

When experimental data cannot be described with linear functions, quadratic response surfaces should be employed to approximate a response function. The full factorial design, central composite design, Box–Behnken design, and Doehlert design are the well‐known second‐order symmetric designs. The central composite design is still used for the development of analytical procedures. But, due to presentation more efficient matrices, the Box–Behnken and Doehlert designs have been recently observed in numerous articles (Bezerra, Santelli, Oliveira, Villar, & Escaleira, 2008).

Drying experiments were carried out with respect to a Box–Behnken design and a central composite design formularized by Design Expert software (DX7) for natural and forced convection runs, respectively. *t*, *RR,* and *Sh* were chosen as response variables, and *IP*, λ, Δ, and V were selected as the main operating variables.

In order to avoid systematic bias, the experimental runs were randomly selected and performed twice and average values of response variables were shown in Tables 1 and 6.

**Table 1:** Experimental design matrix of IR drying of kiwifruit under natural drying air convection

Run	Uncoded variables			Coded variables			*t* (hr)	*RR* (g rehydrated sample/g dried sample)	*Sh* (%)
	*IP* (W)	*λ* (mm)	Δ (mm)	*X*_1_ (W)	*X*_2_ (mm)	*X*_3_ (mm)			
1	2,000	6	700	1	1	0	3.14833	2.3913	77.8564
2	1,500	6	550	0	1	−1	3.60889	2.5	78.009
3	2,000	4	550	1	0	−1	1.71806	2.92857	75.9368
4	2,000	2	700	1	−1	0	1.2	3.66667	72.51
5	1,500	6	850	0	1	1	5.715	2.19048	81.7878
6	1,500	4	700	0	0	0	3.15917	2.65	77.7338
7	1,500	2	850	0	−1	1	2.63444	2.75	77.3787
8	1,500	2	550	0	−1	−1	1.37278	3.57143	74.7105
9	1,000	4	850	−1	0	1	7.34444	2	80.8763
10	1,500	4	700	0	0	0	3.18694	2.4	78.2
11	1,500	4	700	0	0	0	3.13139	2.77	77.1
12	1,500	4	700	0	0	0	3.17917	2.42	77.9
13	2,000	4	850	1	0	1	2.99972	2.58824	78.4539
14	1,000	6	700	−1	1	0	6.46444	1.93103	81.1767
15	1,500	4	700	0	0	0	3.11111	2.78	76.8
16	1,000	2	700	−1	−1	0	2.59917	2.72727	77.6873
17	1,000	4	550	−1	0	−1	3.81806	2.35714	78.0767

To survey the influence of operating variables together with their interactive influences on the response variables, a full quadratic equation is used in RSM as follows (Baş & Boyacı, 2007):

(8) $$y=b_{0}+\sum_{j=1}^{k}b_{j}X_{j}+\sum_{j=1}^{k}b_{\mathrm{jj}}X_{j}^{2}+\sum_{1\leq i<<j}^{k}b_{\mathrm{ij}}X_{i}X_{j}+\varepsilon$$

where y is the response variable; k is the number of variables; *b*_0_, *b_j_*, *b_jj_* and *b_ij_* are regression coefficients of variables for intercept, linear, quadratic, and interaction terms, respectively; and *X_i_* and *X_j_* are the independent coded operating variables. ε is the residual associated with the experiments.

In order to achieve optimal conditions, fitting the Equation (8) to data was done with respect to minimize *t* and *Sh* and maximize *RR*, by the numerical optimization tools of the software. Derringer's overall desirability function (*DF*) is the most often used method in the problem of multicriteria optimization of analytical procedures (Bezerra et al., 2008). *DF* is defined as a weighted geometric mean of *d_i_* as shown by Equation (9) (Chakraborty et al., 2011):

(9) $$DF=\sqrt[{m}]{d_{1}d_{2}\cdots d_{m}}$$

where m is the number of responses investigated in the optimization process.

## 3 RESULTS AND DISCUSSION

### 3.1 Drying kinetics of kiwifruit slices

**Figure 2:**
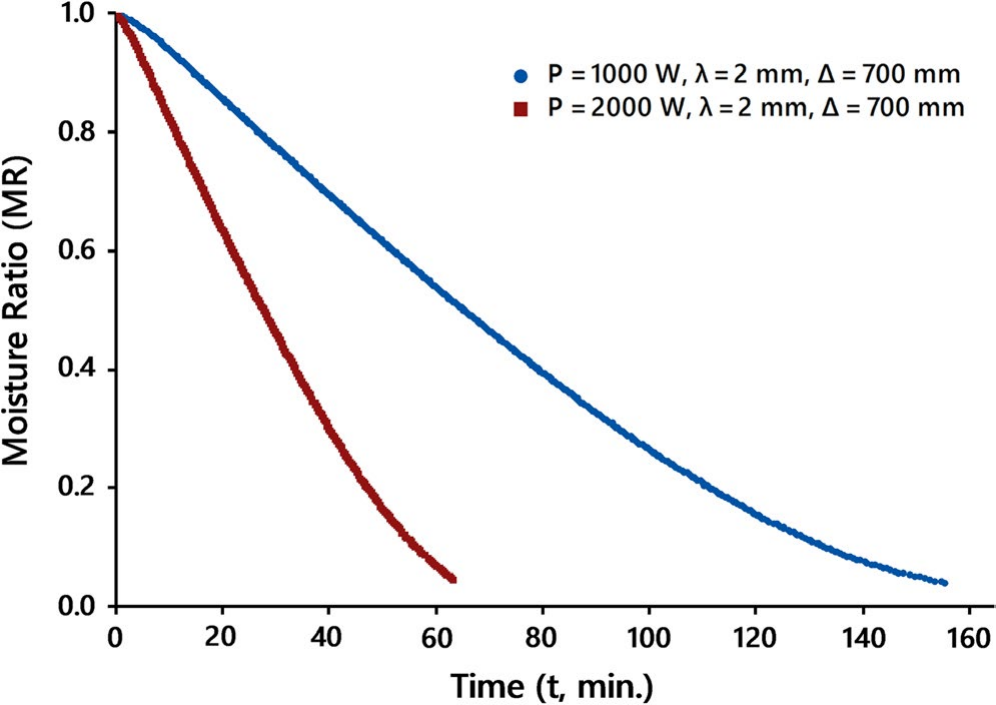
Drying curves of kiwifruit slices at different IR levels, *IP*, at the natural drying air mode

**Figure 3:**
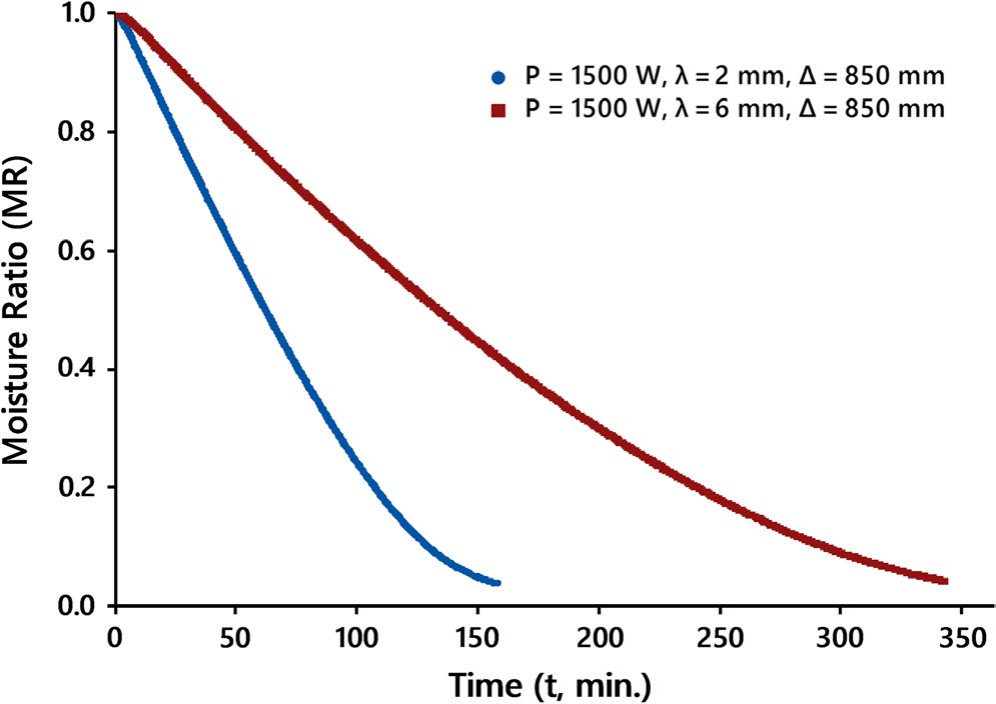
Drying curves of kiwifruit slices at different thickness, *λ*, at the natural drying air mode

**Figure 4:**
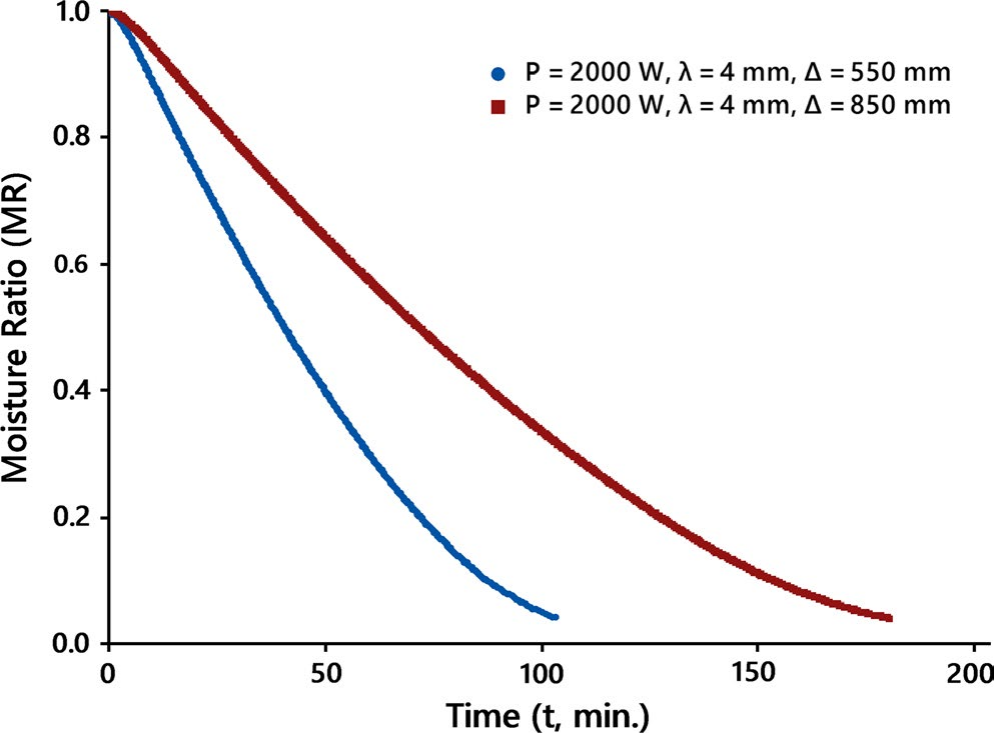
Drying curves of kiwifruit slices at different distances, Δ, at the natural drying air mode

The drying curves of kiwifruit slices undergoing IR drying at various conditions are shown in Figures 2, 3, and 4. Comparison between drying data at various conditions revealed that the drying time of slices at higher IR power or less thickness and/or lower distance was shorter than those of data at the same other options.

This is most likely due to the fact that each of the mentioned items might increase the amount of radiation absorbed by the kiwifruit surfaces, thus describing the higher drying rate during IR drying.

**Figure 5:**
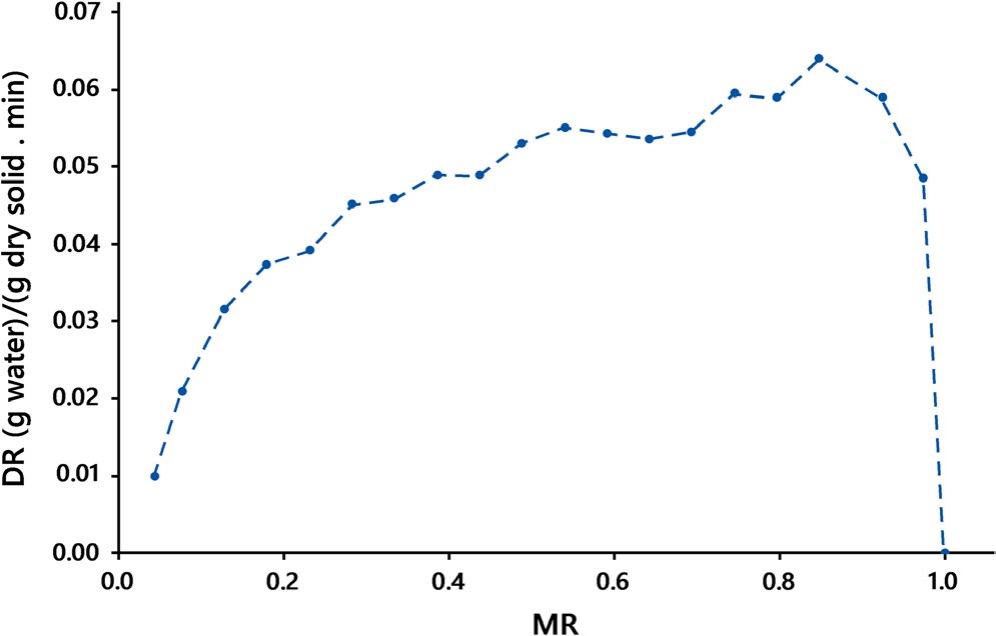
Drying rate of kiwifruit slices at *IP* = 1,000 W, *λ = *2 mm, Δ = 550 mm at the natural drying air mode

**Figure 6:**
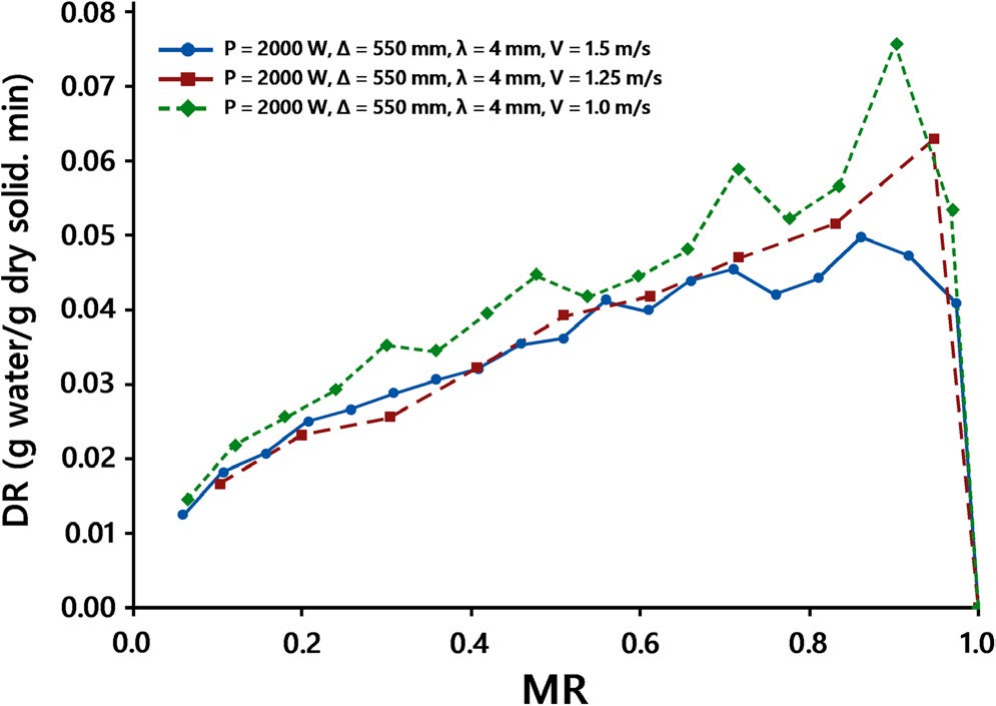
Drying rate of kiwifruit slices at *IP* = 2000 W, *λ = *4 mm, Δ = 550 mm at the forced drying air mode

The variations of the drying rates versus moisture content are shown in Figures 5 and 6. Similar to findings reported by Panyawong and Devahastin (2007), the results suggest that drying rate notably changes with velocity. It is negatively correlated with air velocity. The drying rates were more at the beginning of the drying process, maybe because of evaporation and moisture from the surface of the kiwifruit slices, and then reduced with reducing moisture content, for all the drying conditions when the drying process was governed by moisture diffusion. The accelerated drying rates may be assigned to internal heat generation. The lack of a constant drying rate period can be because of the thin layers of the slice that did not provide a constant supply of moisture, during drying. Also, the drying rate considerably decreases due to some resistance to moisture movement caused by shrinkage (Pathare & Sharma, 2006). An increase in drying rates with an increase in radiation intensity has been observed in other works such as Pathare and Sharma (2006) and Doymaz (2014).

### 3.2 Effects of operating variables on *t*, *RR*, and *Sh*‐natural drying air convection mode

It is obvious that the drying time, *t*, affects the quality of the IR‐dried kiwifruit slices. The rehydration, as an index of quality, is widely employed in the food‐processing industries, denoting the changes in the physicochemical properties of food during drying. These irreversible changes resulted from intense heating and/or lengthened drying will be accompanied by product shrinkage and or lower rehydration values (Tables 1 and 2).

**Table 2:** Summarized statistical data of models for drying time(t), rehydration ratio (RR), and shrinkage (Sh) of IR drying of kiwifruit slices under natural airflow convection

Source	*t*			*RR*			*Sh*		
	*SD*	*R* ^2^	PRESS	*SD*	*R* ^2^	PRESS	*SD*	*R* ^2^	PRESS
Linear	0.60	0.89	9.32	0.19	0.86	0.9	0.75	0.91	13.81
Two‐factor interaction (2FI)	0.48	0.95	10.10	0.19	0.89	1.34	0.78	0.92	25.13
Quadratic	0.31	0.98	10.63	0.17	0.94	1.09	0.72	0.96	38.17

The quadratic model and the linear models are selected as the best regression models for t, RR, and Sh compared with other models, respectively (Tables 2 and 3).

**Table 3:** ANOVA results for drying time (*t*), rehydration ratio (*RR*), and shrinkage (*Sh*) of IR‐dried kiwifruit slices under natural drying air convection

Source	*t*				*RR*				*Sh*			
	Sum of squares	*F* ‐value	*p*‐value		Sum of squares	*F* ‐value	*p*‐value		Sum of squares	*F* ‐value	*p*‐value	
Model	43.38	50.49	<.0001		2.95	26.01	<.0001		72.83	43.02	<.0001	
*X*_1_	15.57	163.11	<.0001		0.82	21.66	.0005		21.32	37.78	<.0001	
*X*_2_	15.49	162.24	<.0001		1.71	45.33	<.0001		34.21	60.62	<.0001	
*X*_3_	8.36	87.54	<.0001		0.42	11.05	.0055		17.30	30.65	<.0001	
*X*_1_ *X*_2_	0.92	9.62	.0173		–	–	–		–	–	–	
*X*_1_ *X*_3_	1.26	13.20	.0084		–	–	–		–	–	–	
*X*_2_ *X*_3_	0.18	1.87	.2140		–	–	–		–	–	–	
$X_{1}^{2}$	0.74	7.72	.0274		–	–	–		–	–	–	
$X_{2}^{2}$	0.20	2.11	.1892		–	–	–		–	–	–	
$X_{3}^{2}$	0.67	6.99	.0332		–	–	–		–	–	–	
*R* ^2^				0.9848				0.8572				0.9085
Adj. *R* ^2^				0.9653				0.8243				0.8874
Pred. *R* ^2^				0.7586				0.7399				0.8278
Adeq. Precision				24.909				16.599				20.310

Analysis of variance (ANOVA) of the best regression models based on collected statistical data such as the highest coefficient of determination value and adequate precision (>4) demonstrates the competency of the selected models. The ANOVA results explain the insignificant terms of model (*p* > .05), which have been omitted from the coded form of final equations as given below (Table 3):

(10) $$t=3.15-1.39X_{1}+1.39X_{2}+1.02X_{3}-0.48X_{1}X_{2}-0.56X_{1}X_{3}+0.42X_{1}^{2}+0.40X_{3}^{2}$$

(11) $$RR=2.62+0.32X_{1}-0.46X_{2}-0.23X_{3}$$

(12) $$Sh\%=77.78-1.63X_{1}+2.07X_{2}+1.47X_{3}$$

where *X*_1_, *X*_2_ and *X*_3_ are the coded values of *IP*, *λ*, and Δ, and, respectively.

It can be seen from the ANOVA results that *X*_1_ and *X*_2_ are the most outstanding factors influencing *t* and *RR*, *Sh*, respectively, followed by other factors.

**Figure 7:**
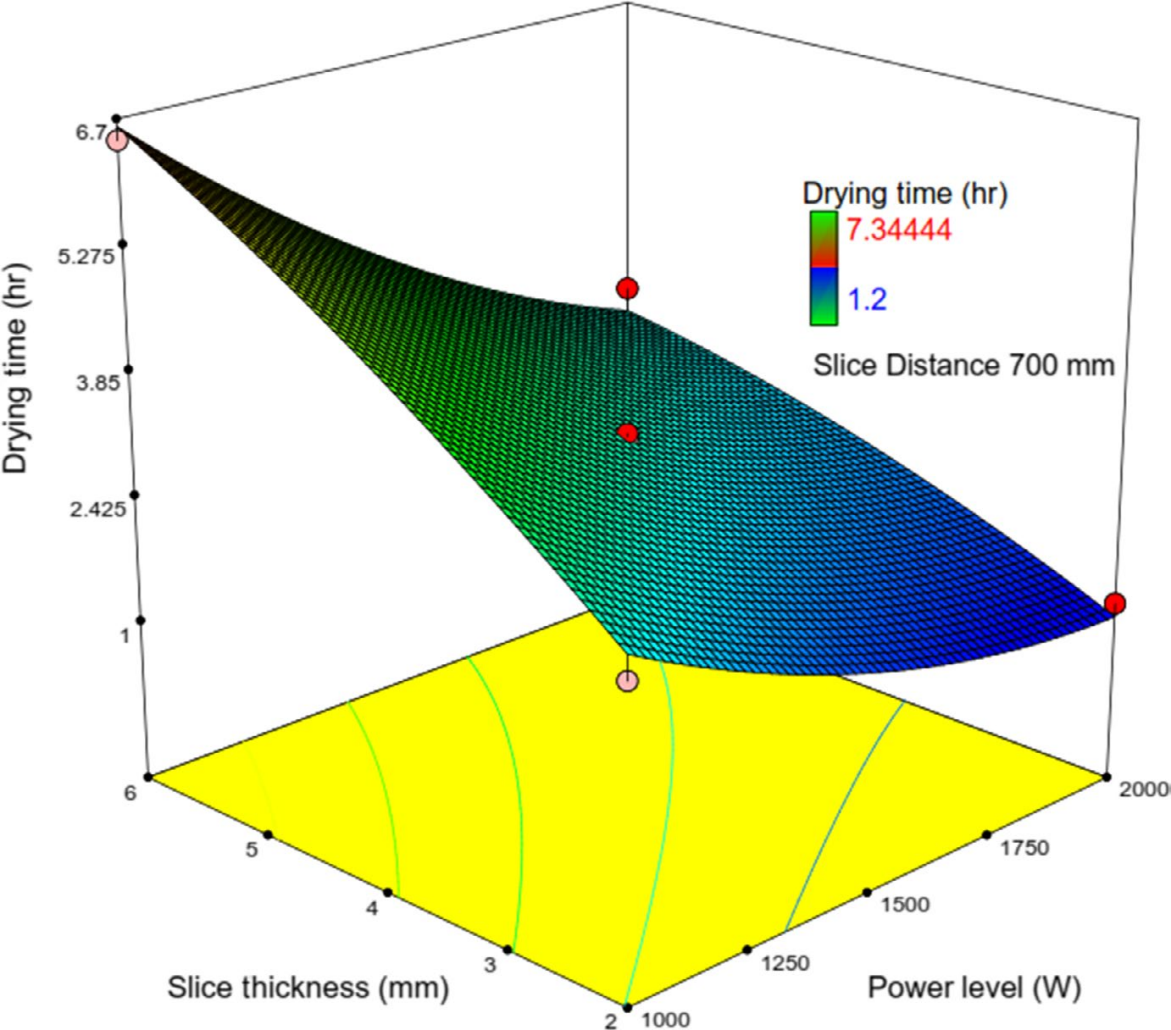
Response surface plot showing the simultaneous effects of *λ* and *IP* on *t*

**Figure 8:**
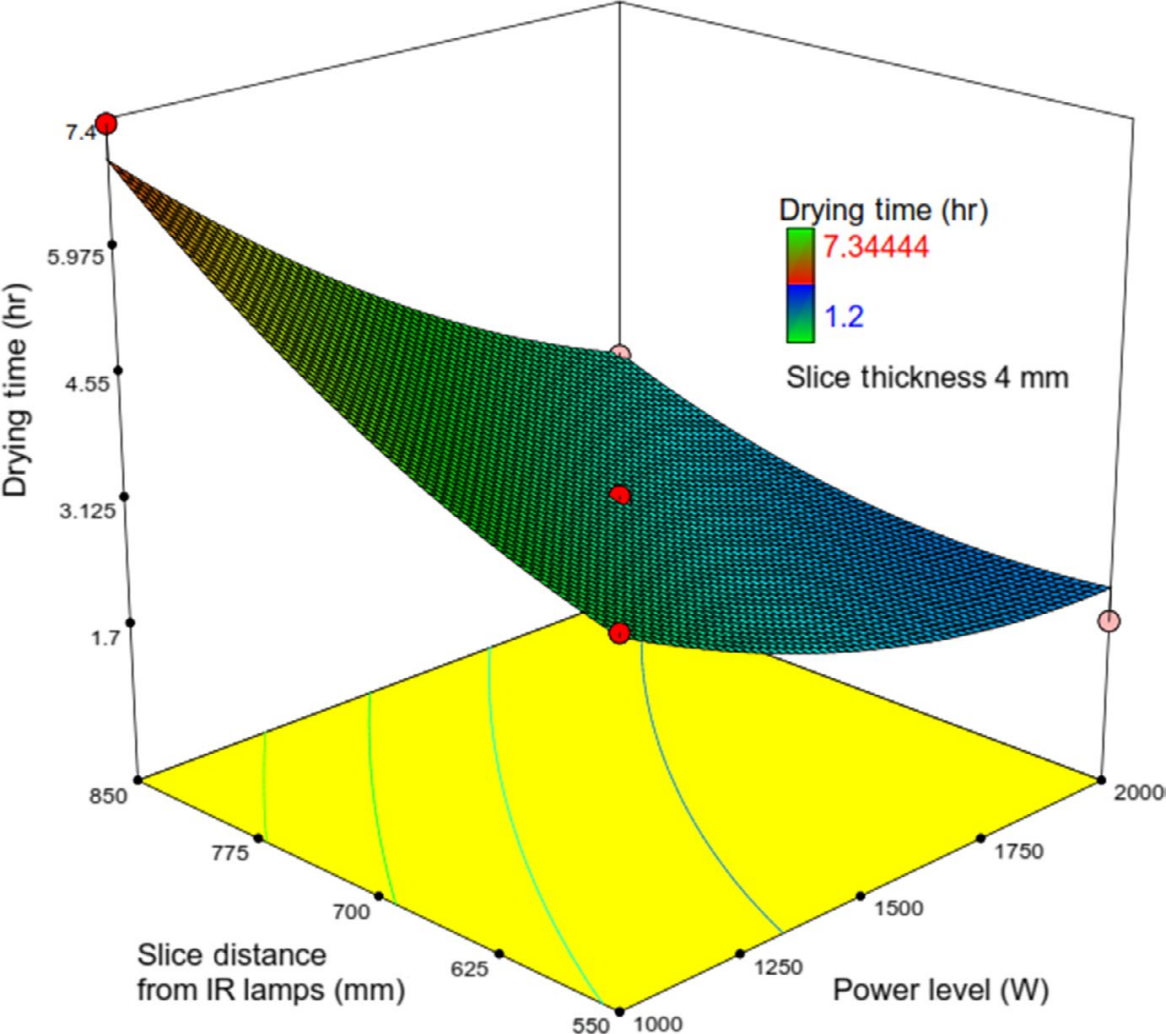
Response surface plot showing the simultaneous effects of *IP* and Δ on *t*

**Figure 9:**
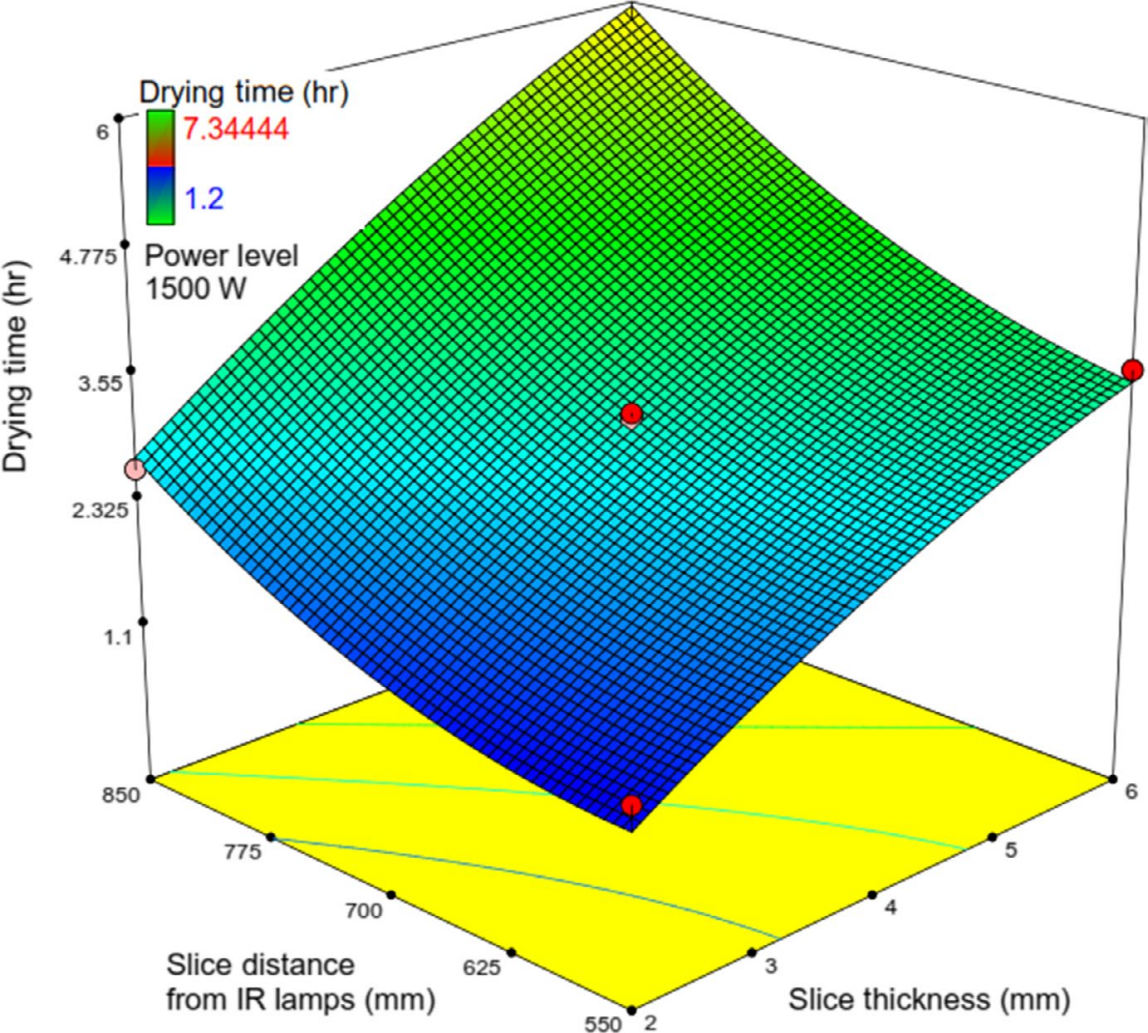
Response surface plot showing the simultaneous effects of *λ* and Δ on *t*

The effect of two factors affecting *X*_2_ and *X*_3_ on *t* can be interpreted as that increasing the conductive resistance and the moisture gradient of the slice due to the increase of *X*_2_ led to the increase in drying times; reducing *X*_3_ caused to obtain a large amount of heat by material and then resulted in excess enthalpy accumulation within it, which was displayed by an increase in product temperature and finally, led to the reduction of drying times. The effect of *X*_1_ on *t* can be assigned to that with an increase in *X*_1_, the extra energy emanated from lamps led to the enhanced slice temperature and drying chamber temperature and finally led to an enhanced drying rate (Das, Das, & Bal, 2009; Sharma, Verma, & Pathare, 2005), and following that resulting in reduced moisture content and drying time of slices(Toğrul, 2005) (Figures 7, 8 and 9).

Figure 7 indicates that at the constant value of *IP* (1,000 W), increasing *λ*, *t* was reached around 6.5 hr. The same incremental manner has been observed at the other values of *IP*. Also, at the different constant values of *λ*, increasing *IP* led to a decrease in *t*. The interactive effects, *IP* versus Δ, and *λ* versus Δ on t have been identified in Figures 8 & 9, respectively.

**Figure 10:**
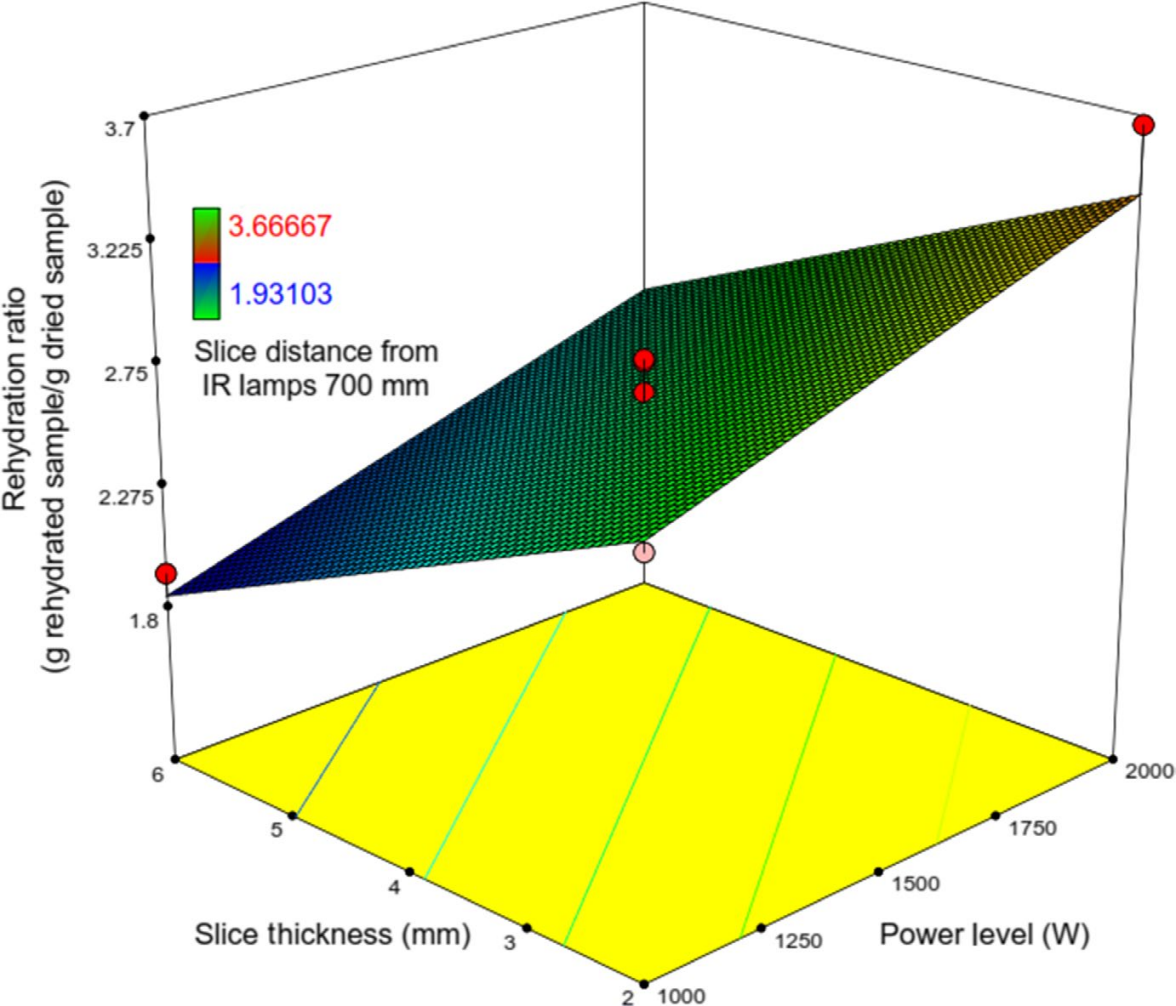
Response surface plot showing the simultaneous effects of *λ* and *IP* on *RR*

**Figure 11:**
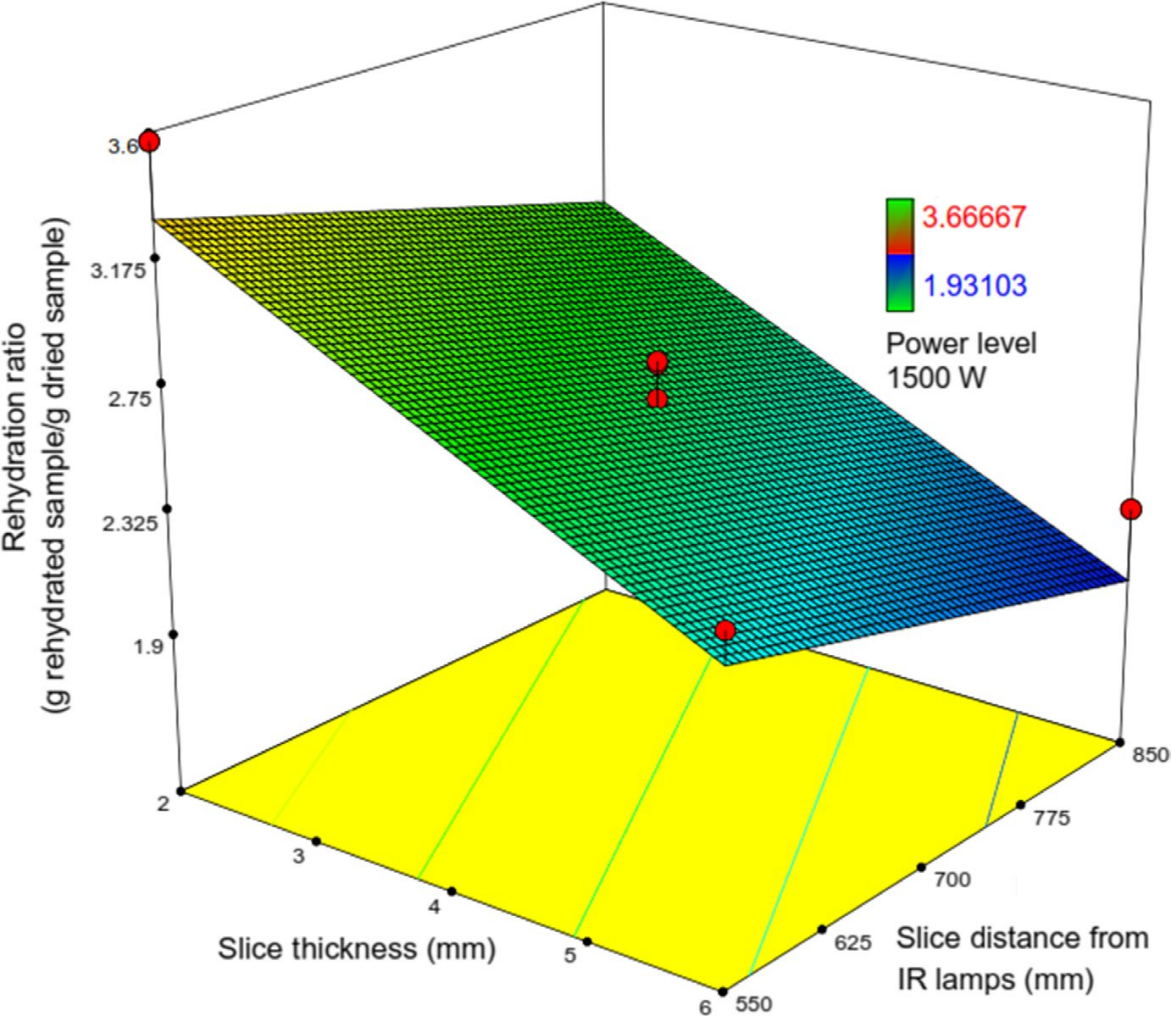
Response surface plot showing the simultaneous effects of *λ* and Δ on *RR*

In general, food containing more moisture content exhibits a greater extent of rehydration (Mongpraneet, Abe, & Tsurusaki, 2002). But rehydration of kiwifruit slices under various conditions of drying process was found in the range of 1.93–3.7 (g rehydrated sample/g dried sample) (Figures 10 and 11).

**Figure 12:**
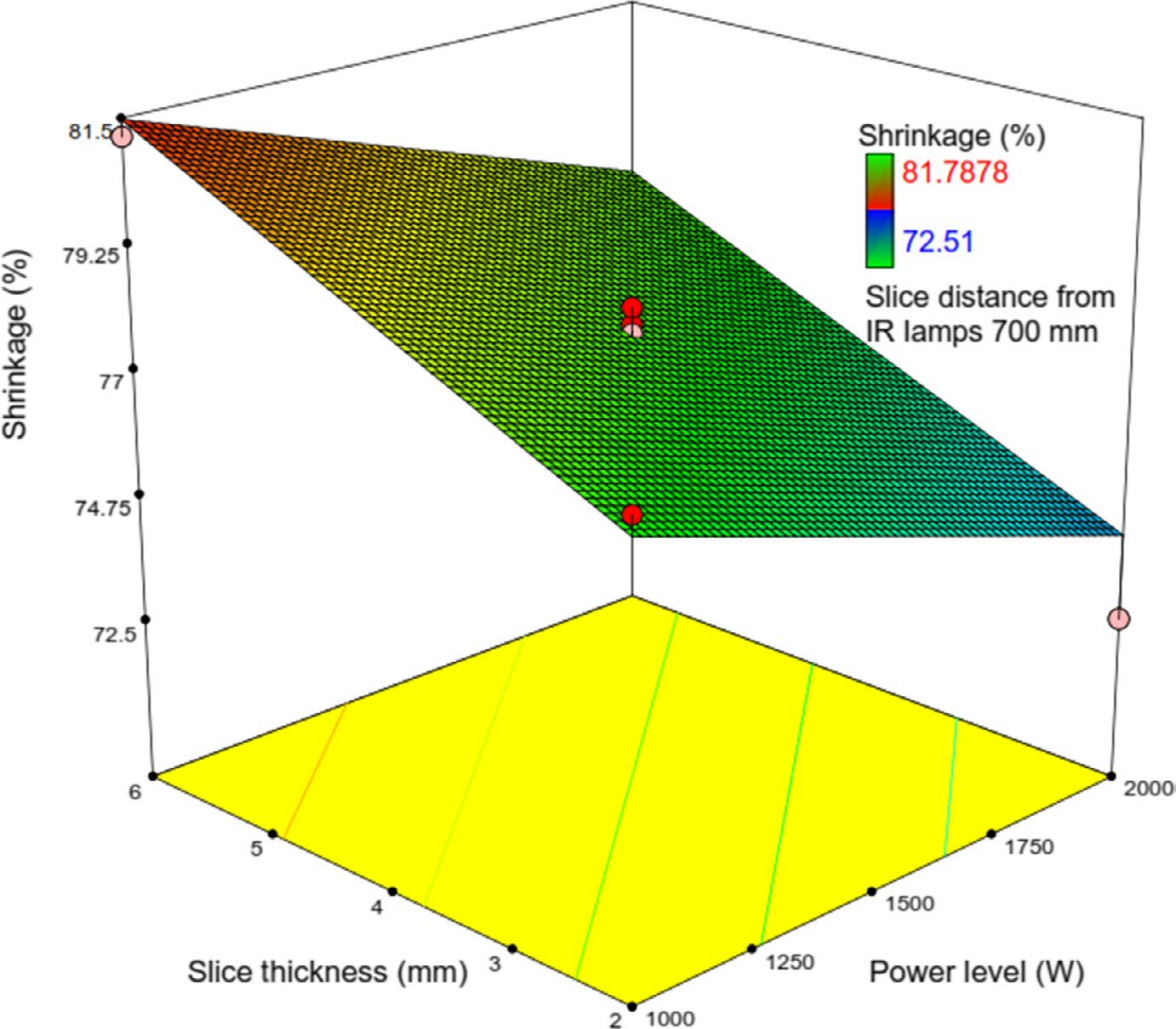
Response surface plot showing the simultaneous effects of *λ* and *IP* on *Sh*

**Figure 13:**
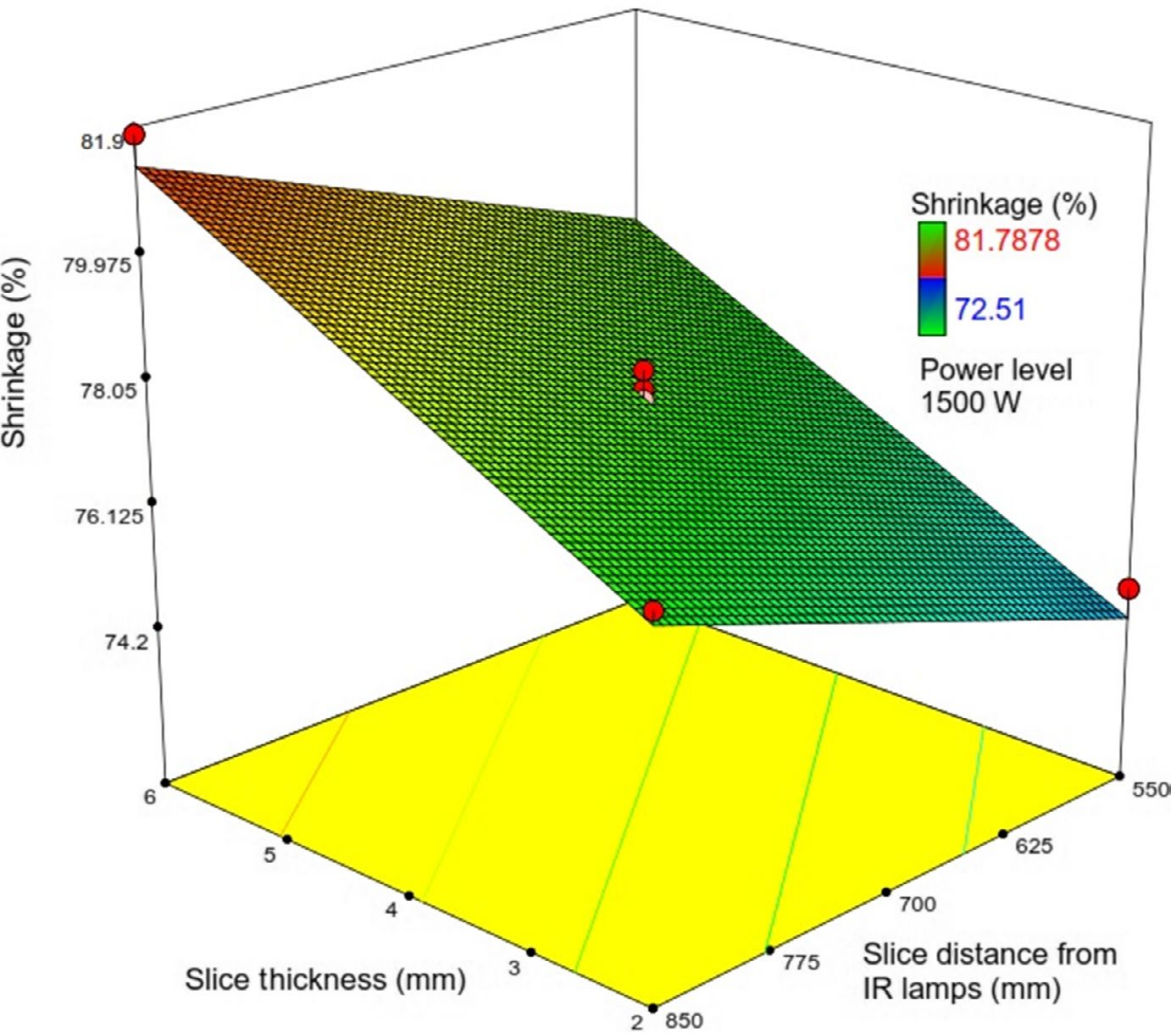
Response surface plot showing the simultaneous effects of *λ* and Δ on *Sh*

Except for *X*_1_, the other operating variables have a reducing effect on *RR*. The effect of *X*_1_ on *RR* can be attributed to the fact that higher *X*_1_ led to dried kiwifruit slices with a relative more porous structure. Similar findings have been observed by Mongpraneet et al. (2002). The increase of *X*_2_ led to reduce rehydration ratio that most likely due to the formation of a little porous medium within dried kiwifruit, thus helping to expedite shrinkage; this corroborated well with the observations of Chakraborty et al. (2011) and Fathi, Mohebbi, and Razavi (2011), and also, similar to the effect of *X*_2_, the increase in *X*_3_ could lead to reduced rehydration ratio. It is observed from Figures 10 and 11 that *RR* is maximized at the lowest values of *λ* and Δ and the highest of *IP* (Figures 12 and 13).

As well, *X*_2_and *X*_3_ have similar effects on *Sh* as evident from Equation (12). Figure 12 shows that at the constant values of *λ*, increasing *IP* would decrease *Sh*. At a given value of *IP*, *Sh* was observed to monotonically increase with increase in *X*_3_ (Figure 13).

The dried foodstuffs under optimal conditions undergo less damage and more rapid rehydration than those of poorly dried (Pan & Atungulu, 2010). Therefore, by maximizing *RR* and minimizing *t* and *Sh* simultaneously, and assigning importance 3 to operating variables, importance 4 to response variables *t* and *Sh* and importance 5 to response variable *RR*, the optimal process conditions are obtained. The optimization program was run and selected the first solution computed by the software with a maximum desirability 0.99 (Table 4). Then, IR drying experiments were done at the assessed optimal conditions and it was identified that the values were predicted to be close enough to the experimental values (Table 5).

**Table 4:** The first solution for optimal conditions in IR drying of kiwifruit under natural drying air convection

*IP* (W)	λ (mm)	Δ (mm)	*t* (hr)	*RR* (g rehydrated sample/g dried sample)	*Sh* (%)
2000	2	550	1.1944	3.63612	72.6053

**Table 5:** Comparison between the predicted and experimental values at optimal conditions in IR drying of kiwifruit under natural drying air convection

Response	Units	Predicted value	Experimental value	Deviation %
*t*	(hr)	1.1944	1.08	−10.59
*RR*	(g rehydrated sample/g dried sample)	3.63612	3.6667	0.834
*Sh*	(%)	72.6053	72.51	−0.131

### 3.3 Effects of operating variables on *t*‐, *RR*‐, and *Sh*‐forced drying air convection mode

After determining the optimum conditions from the experiments under natural drying air convection, *IP* (2,000 W) and Δ (550mm) were fixed for IR drying experiments under forced airflow convection (Table 6).

**Table 6:** Experimental design matrix of IR drying of kiwifruit with forced drying air convection

Run	Uncoded variables		Coded variables		*t* (hr)	*RR* (g rehydrated sample/g dried sample)	*Sh* (%)
	λ (mm)	V (m/s)	*X*_2_ (mm)	*X*_4_ (m/s)			
1	4	1	0	−1	1.87528	2.53009	80.7848
2	2	1.25	−1	0	1.1425	3.34099	78.7585
3	4	1.5	0	1	2.36111	2.84524	83.2491
4	6	1.25	1	0	2.91111	2.10158	83.0153
5	2	1	−1	−1	1.09417	3.50383	75.6343
6	6	1	1	−1	2.74222	2.46159	82.8111
7	4	1.25	0	0	2.03029	2.46491	82.1482
8	4	1.25	0	0	2.01333	2.47447	82.9185
9	4	1.25	0	0	2.04347	2.45748	81.5491
10	6	1.5	1	1	3.06222	2.07243	84.4417
11	2	1.5	−1	1	2.03594	2.90136	79.5418
12	4	1.25	0	0	2.0284	2.46597	82.2338
13	4	1.25	0	0	2.03594	2.46173	81.8915

The next experiments were then carried out with respect to a face‐centered central composite design formularized by the software (Table 6). *λ* and V were chosen as the main operating variables (Table 7).

**Table 7:** Summarized statistical data of models for drying time(t), rehydration ratio (RR), and shrinkage (Sh) of IR drying of kiwifruit slices under forced drying air convection

Source	*t*			*RR*			*Sh*		
	*SD*	*R* ^2^	PRESS	*SD*	R2	PRESS	*SD*	*R* ^2^	PRESS
Linear	0.17	0.93	0.67	0.22	0.78	0.91	1.00	0.85	20.62
2FI	0.14	0.96	0.90	0.22	0.79	1.65	0.98	0.87	35.82
Quadratic	0.11	0.98	0.77	0.20	0.87	2.75	0.55	0.97	12.69

The linear models and the quadratic model are selected as the best regression models for *t*, *RR*, and *Sh* compared with other models, respectively (Tables 7 and 8).

**Table 8:** ANOVA results for drying time(t), rehydration ratio (RR), and shrinkage (Sh) of IR‐dried kiwifruit slices under forced drying air convection

Source	t				RR				Sh			
	Sum of squares	*F* ‐value	*p*‐value		Sum of squares	*F* ‐value	*p*‐value		Sum of squares	*F* ‐value	*p*‐value	
Model	3.80	69.61	<.0001		1.69	18.19	.0005		62.97	41.40	<.0001	
*X*_2_	3.29	120.57	<.0001		1.61	34.74	.0002		44.46	146.15	<.0001	
*X*_4_	0.51	18.65	.0015		0.076	1.64	.2288		10.67	35.08	.0006	
*X*_2_ *X*_4_	–	–	–		–	–	–		1.30	4.26	.0779	
$X_{2}^{2}$	–	–	–		–	–	–		4.91	16.13	.0051	
$X_{4}^{2}$	–	–	–		–	–	–		0.11	0.37	.5602	
R^2^				0.9330				0.7844				0.9673
Adj. *R* ^2^				0.9196				0.7413				0.9439
Pred. *R* ^2^				0.8357				0.5796				0.8051
Adeq. Precision				26.004				12.197				21.648

The ANOVA results demonstrate the competency of the selected models and explain the final equations as given below (Table 8):

(13) $$t=2.11+0.74X_{2}+0.29X_{4}$$

(14) $$RR=2.62-0.52X_{2}$$

(15) $$Sh\%=82.17+2.72X_{2}+1.33X_{4}-1.33X_{2}^{2}$$

where *X*_2_, and *X*_4_ are the coded values of slice thickness and air velocity, respectively.

**Figure 14:**
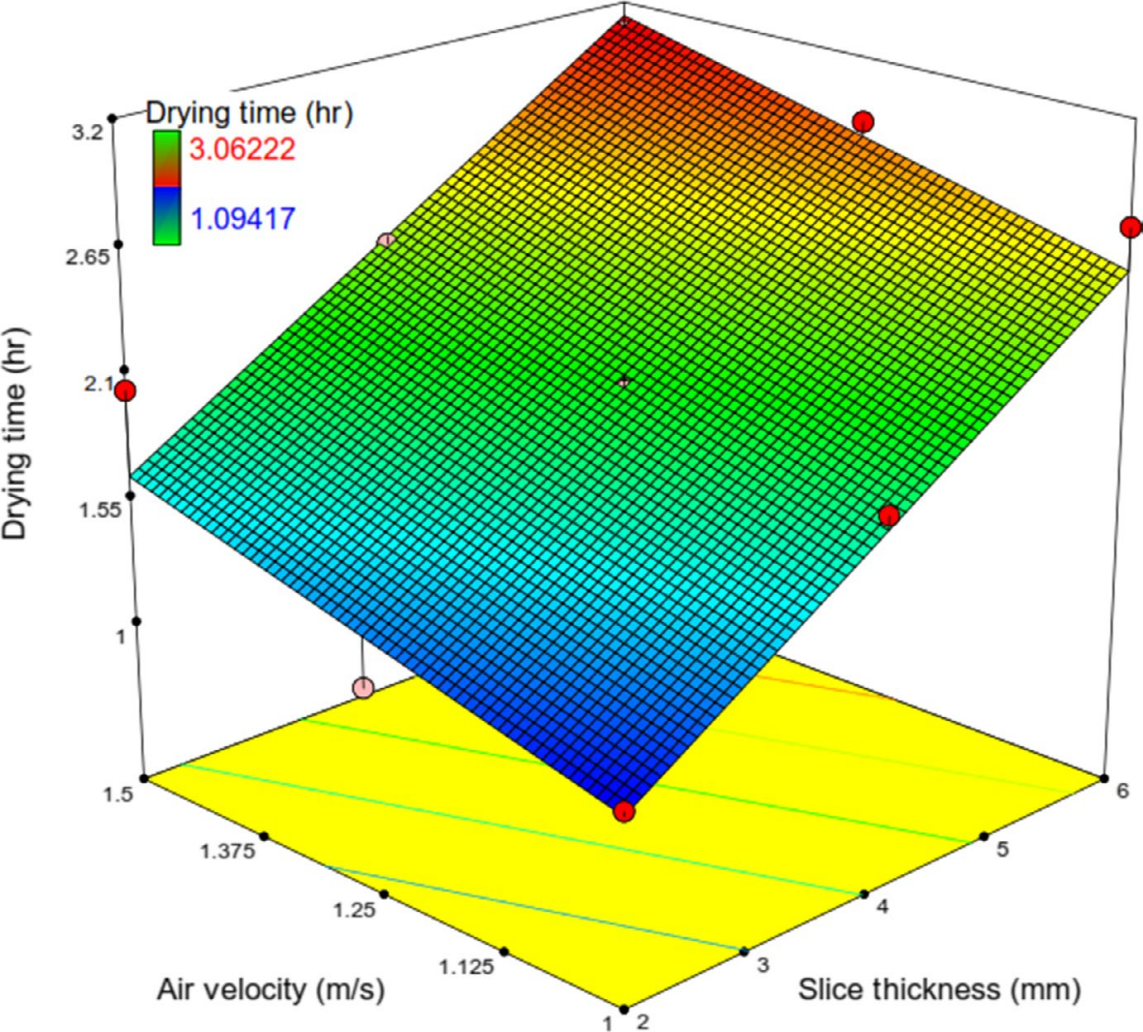
Response surface plot showing the simultaneous effects of *λ* and V on *t*

*X*_2_ is the most outstanding factor, influencing *t* and *Sh*, and only influential factor influencing *RR*. Both operating variables have an increasing impact on *t* and *Sh*. The fact of the significant effect *X*_2_ on *t* and *RR* was mentioned earlier (Figure 14).

It can be attributed to the fact that the increase in air velocity accelerated the cooling effect, which reduced the temperature of the product and water vapor pressure. This led to the enhanced drying time of slices at all drying conditions (Pathare & Sharma, 2006). There is no significant change in the positive direction in the drying time for drying air velocity more than 1m/s (Nowak & Lewicki, 2004; Özdemir et al., 2017). On the contrary, evidence, Equation (13) as well as from the ANOVA (*F* = 18.65), indicates that drying time changes with air velocity. Figure 14 indicates that at the constant values of *V*, with an increase in *λ*, *t* was found to increase.

**Figure 15:**
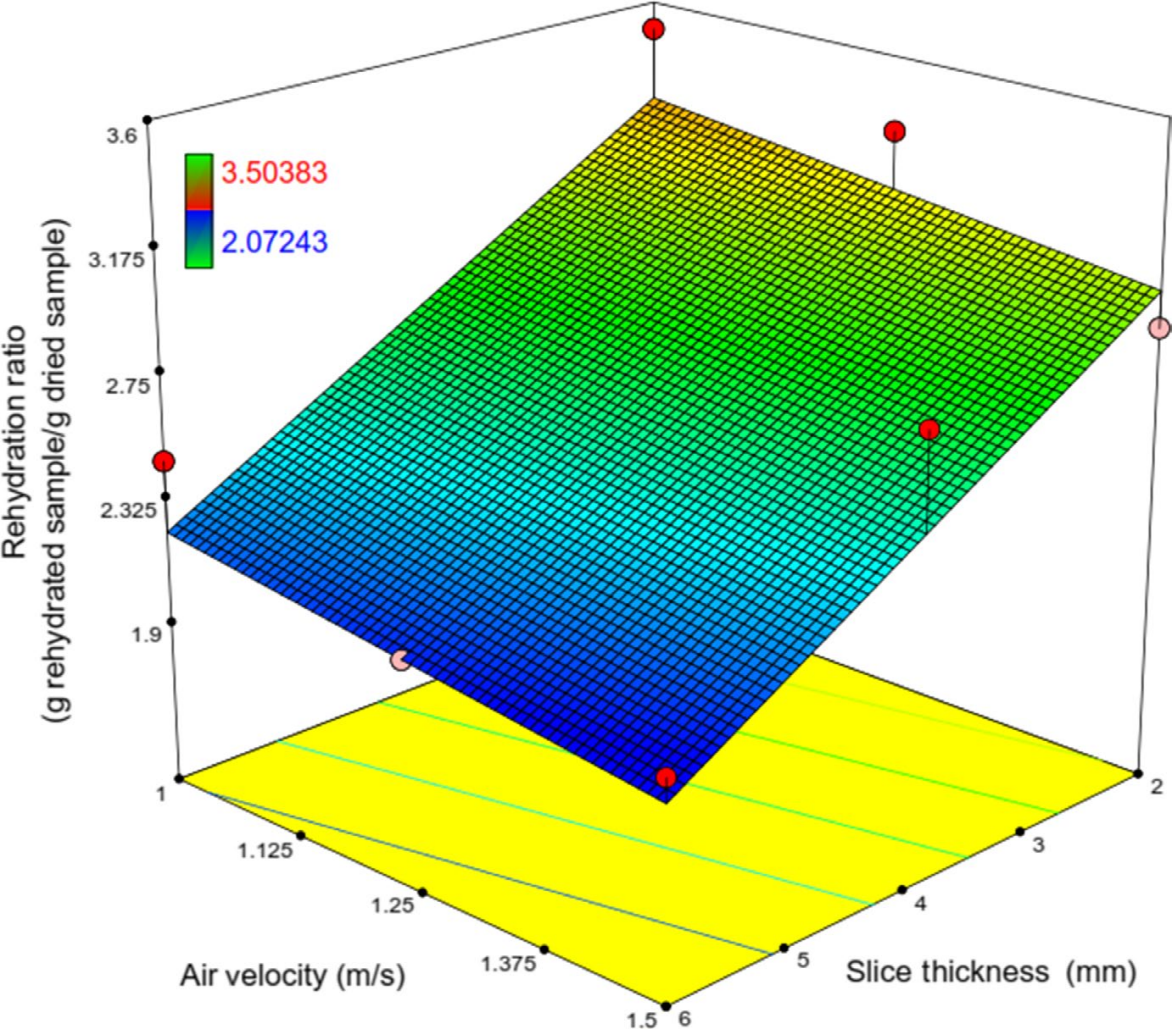
Response surface plot showing the simultaneous effects of *λ* and V on *RR*

But the change in values of *X*_4_ does not affect the amount of rehydration ratio; in other words, there is no significant change in the positive direction in the rehydration ratio for drying air velocity more than 1m/s. This is due to the fact that higher air velocity led to the slight increase in drying time and consequently, resulted approximately in constant *RR* (Figure 15).

**Figure 16:**
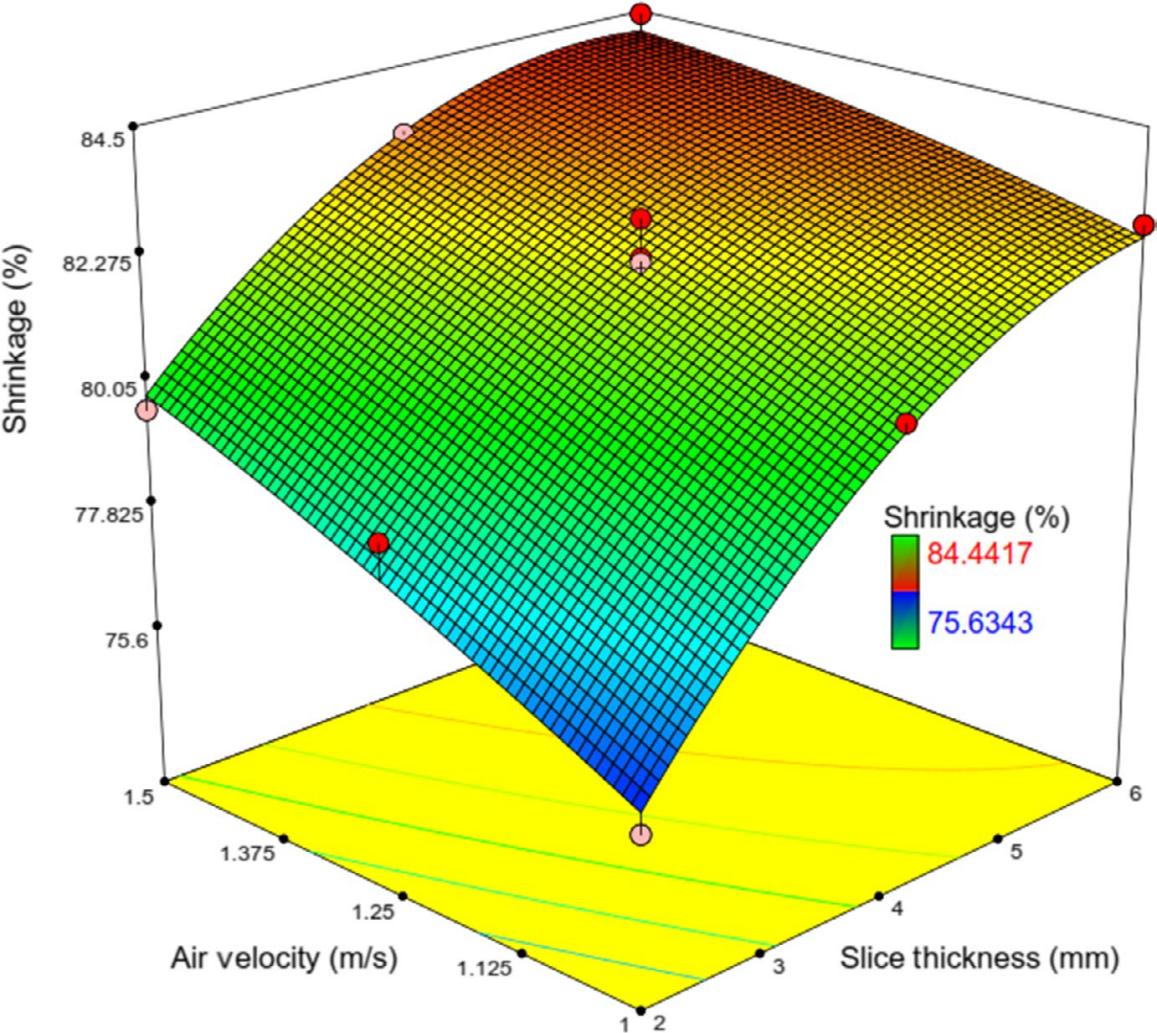
Response surface plot showing the simultaneous effects of *λ* and V on *Sh*

Figure 15 shows that at any constant value of *λ*, an increase in air velocity, almost fixed *RR*. Also, from Figure 15, it can be concluded that *RR* is maximized at the lowest values of *λ* and *V* (Figure 16).

Similar to the effect of *X*_2_, the increase in *X*_4_ could lead to increased moisture content, and as explained earlier, it was because of the prolonged drying time; thus, *X*_2_ and *X*_4_ have the same effects on *Sh*. It can be seen that at any value of *λ*, with a decrease in air velocity, *Sh* was decreased (Figure 16).

Similar to the study of Kocabiyik and Tezer (2009), as air velocity increases, the shrinkage of slices increased. These observations can be explained on the basis of the effect of variables on the mass transfer. In kiwifruit drying, mass transfer occurs with both internal diffusion and external convection control, while the energy transport is externally controlled. Shrinkage may change with the resistance to moisture transfer during drying. Surface resistance dominates at low air velocities; internal stresses inside the slice are at a minimum and moisture profiles are relatively flat. So, at high air velocities, the slice shrinks uniformly and the transfer rate is controlled by internal resistance, especially at low moisture content. The surface of slice becomes drier than the center, so misfit shrinkage is at different points. If air velocities are very high, drying may make the surface of the sample stiff, limiting shrinkage even at the earliest stages. The surface of the sample does not become stiff at low air velocities until the moisture content has attained very low values (Khraisheh et al., 1997) (Tables 9 and 10).

**Table 9:** Optimal conditions in IR drying of kiwifruit under forced drying air convection

Slice thickness (mm)	Drying air velocity(m/s)	Drying time(hr.)	Rehydration ratio (g rehydrated sample/g dried sample)	Shrinkage (%)
2	1	1.0741	3.253	76.01

**Table 10:** Comparison between the predicted and experimental values at optimal conditions in IR drying of kiwifruit under forced drying air convection

Response	Units	Predicted value	Experimental value	Deviation %
*t*	(hr)	1.0741	1.094	1.83
*RR*	(g rehydrated sample/g dried sample)	3.253	3.504	7.16
*Sh*	(%)	76.01	75.634	−0.497

The optimal process conditions were found by assigning the same importance to the variables. The optimization program was selected the first solution computed by the same software with a maximum desirability 0.916 (Table 9). It was identified that the values were predicted to be close enough to the experimental values (Table 10).

## 4 CONCLUSION

Several experiments were done at *IP* (1000–2000 W), *λ* (2–6 mm), Δ (550–850 mm), and V (1–1.5 m/s) based on a Box–Behnken design and a central composite design. Statistical equations generated can predict drying time, rehydration ratio, and shrinkage of IR‐dried kiwifruit as a function of operating variables under natural and forced air convection modes. In all results, slice thickness almost was the most prominent factor, influencing *t*, *RR*, and *Sh*. The operating variables had significant effect on all responses. In IR drying experiments, the optimal conditions were found to be as follows: *IP* (2000 W), Δ (550 mm), and *λ* (2 mm) for natural air convection and V (1 m/s) and *λ* (2 mm) for forced air convection. The drying parameters were optimized based on the quality characteristics of dried kiwifruit and drying time (or energy consumed) during the drying process. At the optimal conditions with regard to a maximum desirability, the lowest values of drying time of the IR‐dried kiwifruit for natural and forced convection were 1.1944 hr. and 1.0741 hr., respectively, which ensures the avoiding excessive consumption of energy from any source.

Besides, the rehydration ratios were 3.636 and 3.253 (g rehydrated sample/g dried sample), which clearly satisfies product quality. Moreover, from the shrinkage of the IR‐dried kiwifruit slices, namely 72.605 and 76.06(%), it was obvious that the IR‐dried kiwifruit slices had become more stable compared with fresh samples.

It is worth mentioning the developed statistical multivariate model equations can be very useful for the design and operation of IR dryer when there is no certain multidimensional IR drying model for high‐value foodstuffs such as kiwifruit.

## CONFLICT OF INTEREST

The authors declare that they have no conflict of interest.

## ETHICAL APPROVAL

This study does not involve any human or animal testing.

## INFORMED CONSENT

For this type of study, formal consent is not required.
